# Marginal fit of crowns fabricated via simultaneous digital workflows for integrated fiber post-and-core and crown: an in vitro study

**DOI:** 10.1186/s12903-026-08732-6

**Published:** 2026-06-03

**Authors:** Xiaoyang Guo, Yundeng Xiaowen, Maomao Gao, Yixuan Fu, Zengbo Zhao, Zhiyu Chen

**Affiliations:** 1 Department of Prosthodontics, Hospital of Stomatology Hebei Medical University, Hebei Key Laboratory of Stomatology, Hebei Clinical Medical Research Centre for Oral Diseases, 383 Zhongshan East Road, Chang’an District, Shijiazhuang, 050017 China; 2 Department of Oral Emergency, Hospital of Stomatology Hebei Medical University, Hebei Key Laboratory of Stomatology, Hebei Clinical Medical Research Centre for Oral Diseases, Shijiazhuang, China

**Keywords:** CAD-CAM, Crown fit, Post-and-core, Micro CT

## Abstract

**Background:**

For teeth with extensive structural defects, the post-and-core crown is a well-established, but multi-visit approach to preserving the natural tooth. Digital workflows enable simultaneous fabrication of the post-and-core and crown; however, the marginal fit of the crown produced by these workflows—a critical indicator of clinical performance—has not been systematically assessed. This in vitro study aimed to compare crown fit between two simultaneous digital workflows and a conventional sequential method.

**Methods:**

Thirty intact mandibular first premolars were randomly assigned to three groups (*n* = 10): traditional step-by-step workflow (TSS group), digital cast splicing method (DSM group), and physical polyether cast (PPC group). All restorations were fabricated using computer-aided design and manufacturing (CAD-CAM) and subsequently cemented. Marginal and internal fit were assessed using microcomputed tomography (micro-CT). The adhesive volume and surface area of the abutment teeth were quantified three-dimensionally to calculate the average intracoronary cement thickness (AICT). The crown fit was evaluated in two dimensions at 16 locations, including the marginal gap (MG), axial wall gap (AWG), axial occlusal intersection gap (AOG), and occlusal gap (OG). Statistical analyses were conducted using appropriate parametric or non-parametric tests with a significance level of α = 0.05.

**Results:**

No significant differences were observed among the three workflows for AICT (*P* = .083) and AOG (*P* = .204). Significant inter-group differences were found for MG (*P* = .022), AWG (*P* = .004), and OG (*P* < .001) regarding the 2D parameters. Specifically, the DSM group exhibited a larger MG than the TSS and PPC groups; the PPC group demonstrated a larger AWG than the TSS and DSM groups; and the TSS group showed a larger OG than both the PPC and DSM groups. Despite these statistical differences, all measured values remained within the 120 μm threshold.

**Conclusion:**

Simultaneous digital fabrication of integrated post-and-core crowns achieved a clinically acceptable marginal fit. Within the limitations of this in vitro study, digital workflows represent a reliable alternative to conventional multi-visit protocols for the fabrication of integrated post-and-core and crowns.

**Trial registration:**

This study involves ex vivo experiments using extracted teeth (biological samples) and does not qualify as a clinical trial. Therefore, clinical trial registration does not apply to this study.

## Background

For endodontically treated teeth with compromised coronal structure, post-and-core restoration provide essential retention and structural support [1]. The clinical success depends on several critical factors, including a ferrule height of at least 2 mm, a crown-to-root ratio exceeding 0.9 [2, 3], and a post diameter approximately one-quarter of the root diameter [4]. With a 5.3-year survival rate of up to 98.6% [5], post-and-core crowns offer advantages over implant-supported prostheses, such as shorter treatment time, preservation of the residual root, and superior gingival aesthetics [6]. Traditionally, the post-and-core restoration is fabricated separately from the crown, requiring two laboratory procedures and three clinical appointments—increasing microleakage risk and compromising patient experience [7].

Post-and-core materials include prefabricated fibers, cast metals, and CAD-CAM zirconia, each with limitations such as poor fit, high risk of root fracture, or prolonged fabrication time [8–10]. Emerging materials like polyetheretherketone (PEEK) and fiber-reinforced composite resin offer promising alternatives [11, 12]. CAD-CAM glass fiber posts, in particular, exhibit bond strength superior to PEEK [13, 14], an elastic modulus similar to dentin, and favorable fracture resistance and adaptation [15], while eliminating the need for post-processing surface treatment [16]. These advantages, combined with digital workflows, formed the basis for this study. A half-digital technique collected intracanal profile data by optically scanning the conventional post-space impression [17, 18] or the acrylic resin or wax patterns of the post-and-core [19], facilitating the design of a customized post shape that matches the root canal. The feasibility of fully intraoral digital scanning for post-and-core impressions [20], as reported in certain studies, remains primarily restricted to wider root canals and depends on the scanning depth capabilities of intraoral scanners [21], which may limit its application in cases with narrower canals or complex anatomical features.

While the conventional method sequences the post-core and crown fabrication, Ju-Hyoung Lee pivoted by utilizing an existing crown to guide the core fabrication via a milled matrix [22]. By applying the reverse-engineering approach to the digital domain and utilizing millable fiber posts, our group conceptualized and realized a logical progression: the simultaneous digital design and fabrication of the crown and its supporting post-and-core foundation. The first method involves a virtual alignment technique, which has been preliminarily reported in a case study [23]; the second method utilizes a polyether pattern [24] that replicates both the prepared tooth and the post space. This pattern is digitally scanned in two stages—first for crown design, and then, after removal, for post-and-core design—enabling the simultaneous CAD-CAM fabrication of both restorations. Utilizing digital synchronization is designed with the objective of optimizing the treatment workflow and enhancing efficiency by reducing clinical sessions. However, the feasibility of these two innovative digital workflows for integrated post-and-core and crown fabrication has not been systematically evaluated. Given that the marginal and internal fit of the crown restoration is a paramount determinant of long-term success and durability [25, 26], these parameters were employed as outcome indicators to assess and compare the performance of the two workflows. Accordingly, this study aimed to evaluate the two digital protocols by focusing on the marginal and internal fit of the crowns, without assessing the fit of the post-and-core portions.

Micro computed tomography (µCT) is a non-destructive visualization technique that is employed to evaluate the fit of restorations, enabling assessment of marginal and internal gap at multiple sites in both two-dimensional (2D) and three-dimensional (3D) views [27]. Although µCT has been used to assess adhesive volume [28–30], comparisons are complicated by variations in tooth morphology and preparation design. While a clinically accepted threshold of 120 μm exists for 2D marginal and internal gaps measurements [31], no standardized criteria have been established for 3D volumetric assessment of the cement layer. The existing literature on cement volume largely relies on qualitative inter-group comparisons, which are highly sensitive to confounding factors such as cement space settings and tooth morphology [28]. This study introduces a novel and comprehensive evaluation protocol to overcome this limitation. We propose a 3D volumetric averaging method to quantify the total cement space, enabling a comprehensive assessment of crown adaptation. This method incorporates focused 2D measurements at four clinically significant sites [32] to assess localized fit against established clinical standards.

This dual approach combines global volumetric assessment with localized morphometric analysis to achieve a thorough evaluation of crown fit. The null hypothesis was that there would be no significant difference in the marginal and internal fit of the final crowns fabricated using the two digital workflows for simultaneous fabrication of integrated fiber post-and-core with CAD-CAM crowns and the traditional workflow.

## Materials and methods

**Fig. 1:**
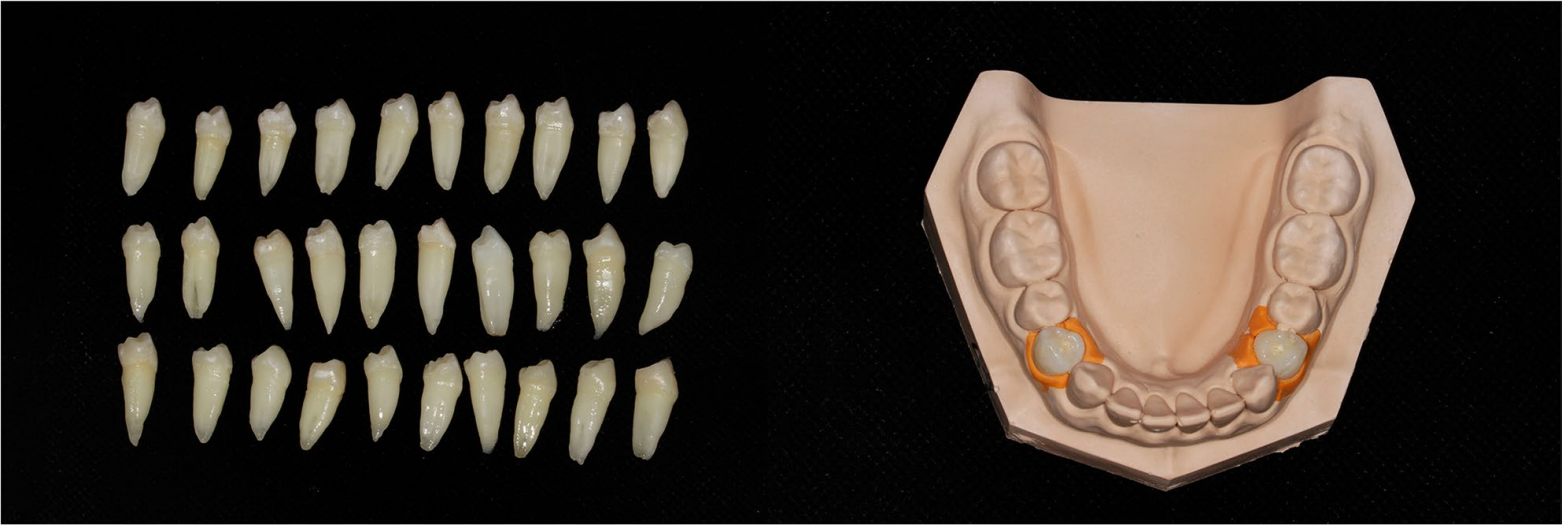
Isolated teeth and standard mandibular plaster cast

Thirty intact single-rooted human mandibular premolars were selected for this study. All specimens demonstrated fully developed apices and were devoid of had cracks, caries, restorations, abrasion, erosion, or fractures. The dental roots were wrapped in light-body polyvinyl siloxane (Silagum Intro Kit C; DMG, Hamburg, Germany) to simulate an artificial periodontal ligament. Then, they were embedded in a standard gypsum mandibular dental arch cast (Royal Rock; Pemaco, St. Paul, MN, USA). Each gypsum cast included two extracted teeth, for a total of 15 casts (Fig. 1). The crowns were standardized by sectioning them 2 mm occlusal to the gingival margin with a water-cooled rotary instrument and a high-speed dental handpiece (M; Mani, Utsunomiya, Japan). Subsequently, each tooth underwent endodontic treatment [33], followed by preliminary crown preparation. A dentin ferrule measuring 1.5 mm in height and 1 mm in thickness was prepared, along with a round-shouldered finishing line at the gingival level with a width of 0.8 mm. This dimension was selected as it represents a clinically common and acceptable minimum threshold, thereby providing a clinically relevant and conservative test condition for evaluating the restoration’s performance [16]. The root canal post space was prepared with reamers (Pesso; Ivoclar, Schaan, Liechtenstein) to simulate clinical practice, ensuring at least 5 mm of the apical seal remained intact and that the post length exceeded half of the total root length. By using a lottery method, the teeth were randomly assigned into three groups (*n* = 10) for different workflows [28]. Specifically, each specimen was assigned a unique identifier (1–30), and corresponding slips were placed into opaque, sealed envelopes. An independent operator not involved in specimen preparation or subsequent measurements conducted the draw. Based on the drawn sequence, specimens were allocated equally (*n* = 10 per group) to ensure concealed and unbiased group assignment [34].

**Fig. 2:**
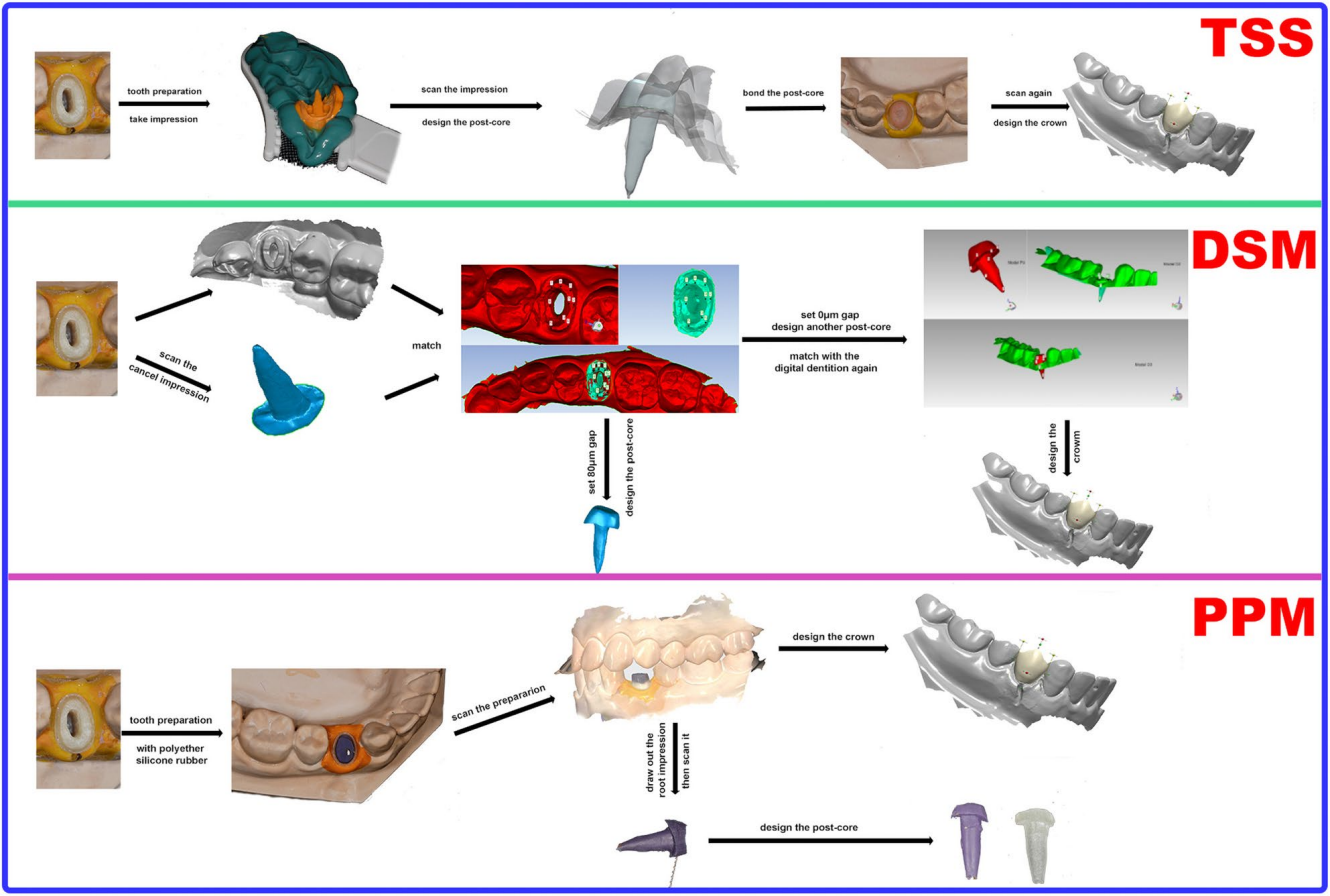
Stepwise workflows of 3 post-and-core crown production methods

The traditional step-by-step workflow (TSS) (Fig. 2) used a polyvinyl siloxane impression (Honigum MixStar Mono; DMG, Hamburg, Germany) of the post space, made with a sectioned plastic impression tray (Premium Plus 3-in-1; Kerr, Orange, CA, USA). The impression included the target tooth, the post space, the coronal residual dentin, and the adjacent gypsum-cast teeth on each side. The impression was then scanned with a laboratory scanner (3Shape D700; 3Shape, Copenhagen, Denmark) to obtain a virtual cast, which was used to design a digital custom post-and-core through a CAD software program (3Shape Dental System; 3Shape, Copenhagen, Denmark). The cement was set to 80 μm. A custom integrated fiber-reinforced composite post-and-core was milled from composite resin blocks (cylindrical fibrous resin block; Eurasia Ruikang New Material Technology Co., Tianjin, China) using a robotic milling machine (Ideal Mill 5 A; Baden Technology, Beijing, China). After adjustment and seating, the post-and-core was cemented with a dual‑cure resin cement (Panavia F2.0; Kuraray Noritake Dental, Tokyo, Japan) strictly following the manufacturer’s instructions. The posts were polymerized using an LED curing light (Valo Cordless; Ultradent Products, South Jordan, UT, USA) for 40 s on each axial wall of the tooth. Then, the tooth preparation was completed. A digital scan of the abutment and the dental arch was obtained using an intraoral scanner (TRIOS 3; 3Shape, Copenhagen, Denmark) to design a crown with an intended internal gap of 50 μm. The crown was milled from a composite resin block (Brilliant Crios; Coltène, Altstätten, Switzerland) using a milling machine (VHF Z4; Camfacture, Altstätten, Switzerland). Finally, the crown was cemented onto the abutment after the necessary adjustments using a dual‑cure resin cement (Panavia F2.0; Kuraray Noritake Dental, Tokyo, Japan). Prior to cementation, the intaglio surface of the crown and the abutment surface were treated according to the manufacturer’s instructions. The crowns were seated on their respective dies with firm finger pressure [35], and excess cement was carefully removed. Subsequently, the resin cement was light‑cured for 40 s per surface (mesial, distal, buccal, lingual) using an LED curing unit (Valo Cordless; Ultradent Products, South Jordan, UT, USA) with an output irradiance of 800 mW/cm².

**Fig. 3:**
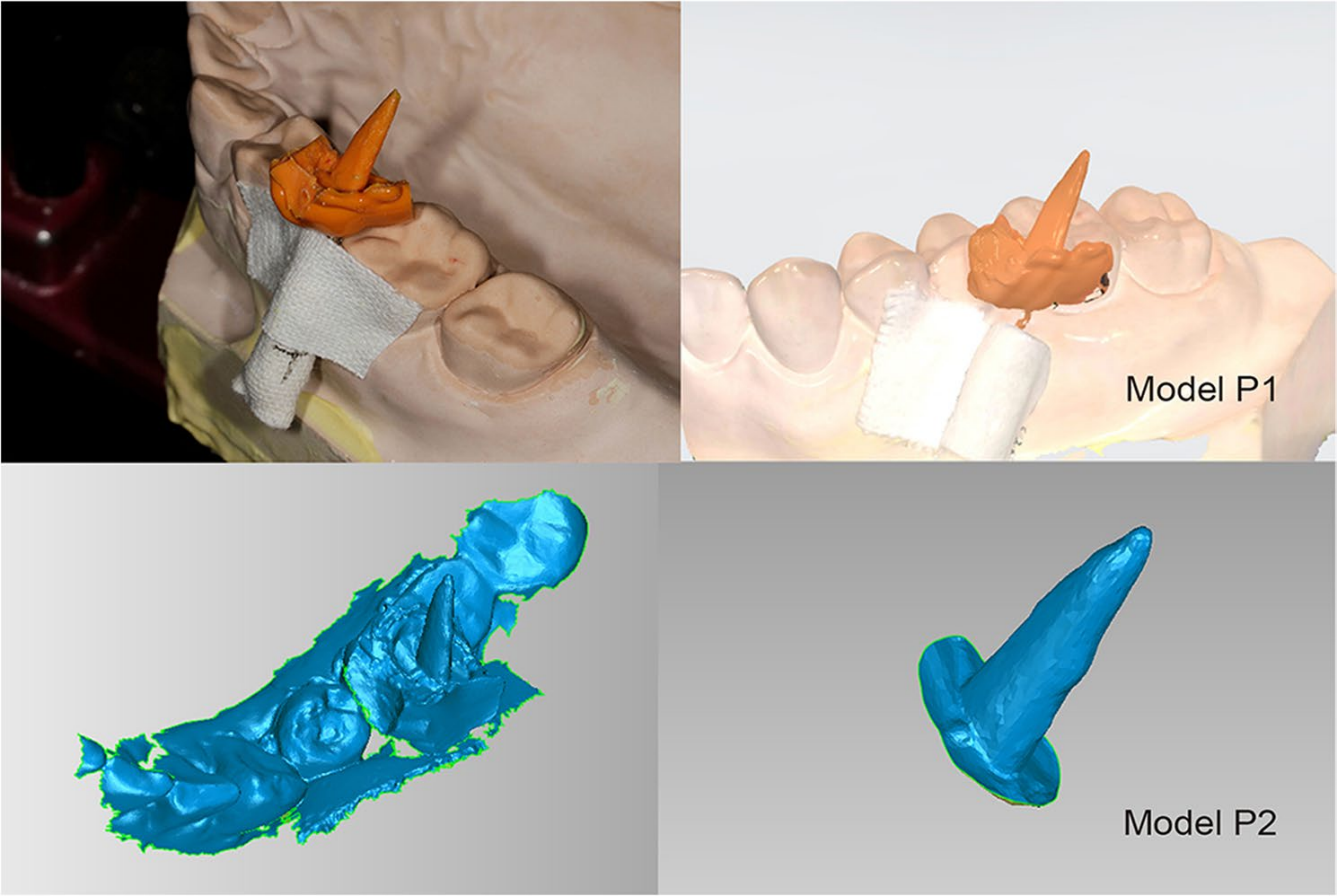
Acquisition of post space impression by using Cast P1 and Cast P2

**Fig. 4:**
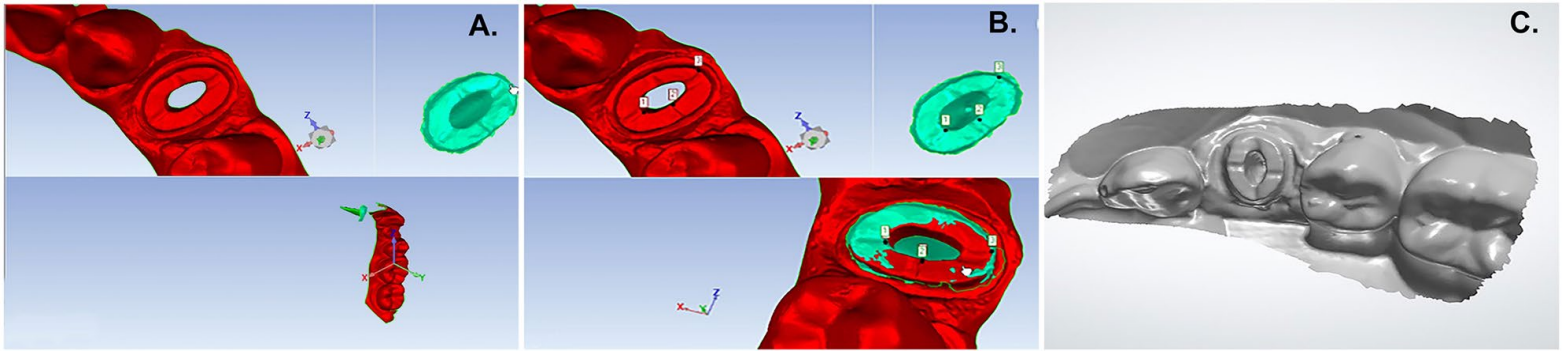
Superimposition of root canal shape onto digital dentition cast. **A**, Importing digital casts into software. **B**, Harmonizing coordinate systems through point selection. **C**, Combining data into digital dentition cast

**Fig. 5:**
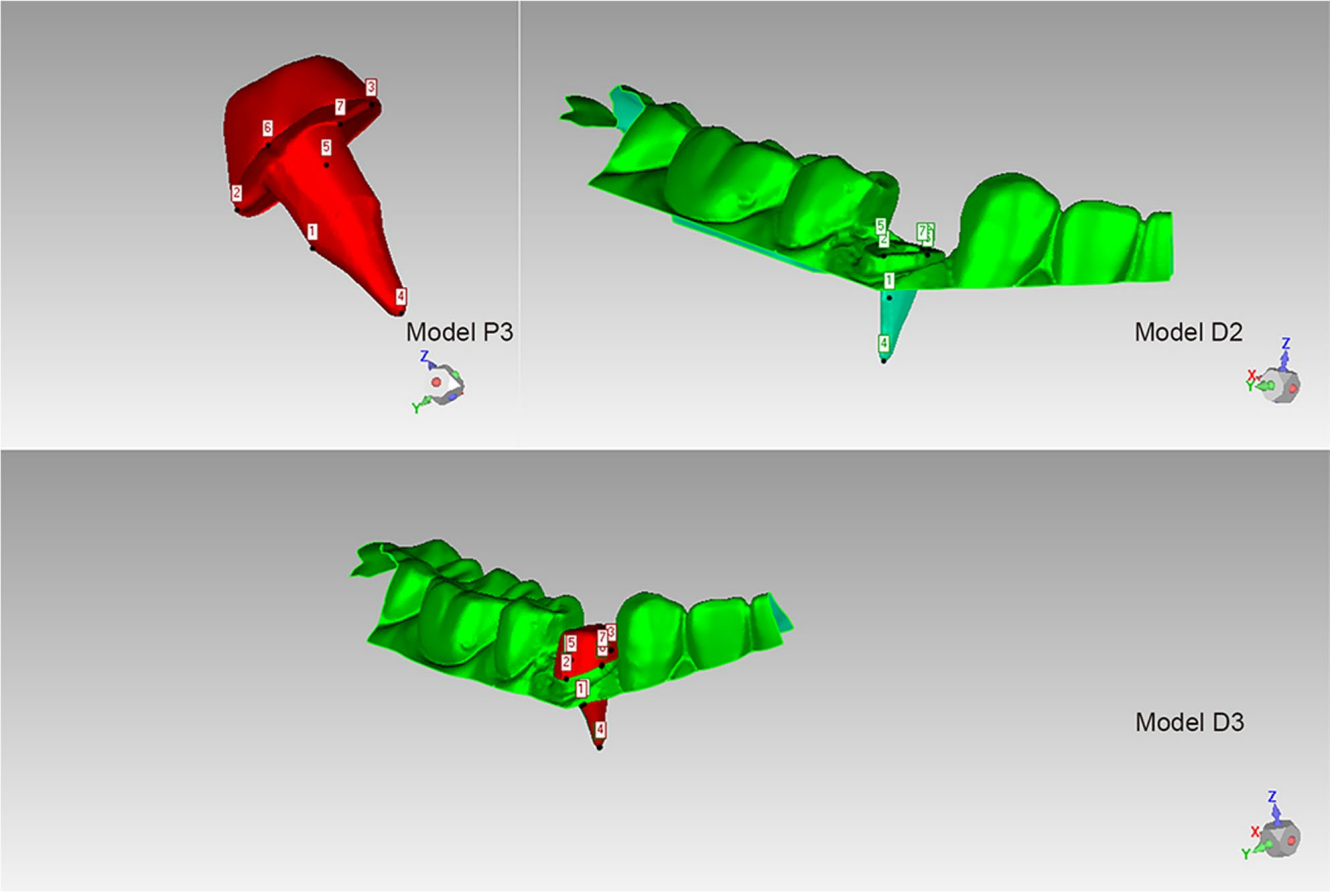
Schematic illustration of the superimposition process for obtaining D3

The digital cast splicing method (DSM) was performed using 3D modeling software (Fig. 2). Four transverse grooves were made on the tooth’s cross-section to serve as landmarks for virtual cast alignment. Light-body polyvinyl siloxane (PVS) (Silagum Intro Kit C; DMG, Hamburg, Germany) was delivered into the root canal until the post space was filled and the abutment was completely covered. A #40 K-file (Dentsply Sirona, York, PA, USA) was then inserted into the root canal to reinforce the PVS. Following polymerization, the PVS impression (together with the K-file) was carefully removed and placed onto the gypsum cast, ensuring that the coronal part of the PVS achieved intimate contact with the cast surface [36]. The combined assembly (PVS and cast) was digitized using an intraoral scanner (TRIOS 3; 3Shape, Copenhagen, Denmark), and the acquired 3D dataset was saved as Cast P1 (Fig. 3). In a software program (Geomagic Wrap 2017; 3D Systems, Rock Hill, SC, USA), Cast P1 was cropped to extract post-space data, which was named Cast P2 (Fig. 3). The abutment tooth was separately scanned using the intraoral scanner (TRIOS 3; 3Shape, Copenhagen, Denmark) to generate Cast D1. Due to the identical root canal orifice data of Casts D1 and P2, they were aligned to create an integrated Cast D2 (Fig. 4), a virtual dental arch with abutment and post-space data. Similar to the TSS group, Cast D2 was used to digitally design two virtual models of the custom post-and-core, referred to as Cast P3 and Cast Post, with 0 μm and 80 μm cement spacers reserved, respectively. The coordinate systems of Casts D2 and P3 were unified and subsequently merged to form Cast D3, mimicking the post try-in process. Finally, a crown was digitally designed based on Cast D3 (Fig. 5), and a custom integrated post-and-core was milled using Cast Post.

A stability validation test was performed to evaluate the reproducibility and precision of digital superimposition in the DSM workflow. The Cast D1 and Cast P2 were superimposed 5 separate times by the same operator, generating 5 complete digital arch models (Cast D2a to Cast D2e). Subsequently, these 5 models were pairwise aligned using a “best-fit alignment” algorithm, obtaining a total of 10 alignment instances. For each pairwise alignment, the root mean square (RMS) error, representing the average surface deviation between the 2 models, was recorded. The RMS deviation values (*n* = 10) underwent statistical analysis. The Shapiro–Wilk test showed that the data did not deviate significantly from a normal distribution (*P* = .159; Table 1). The mean RMS error for all alignments was 20 μm, which is well within the acceptable range of 10–50 μm reported for similar digital workflows in the literature [37]. The high reproducibility of the digital superimposition protocol (as indicated by low standard deviations) confirms the consistency of the fit measurements, providing a solid foundation for comparing the three workflows.

**Table 1:** RMS estimate value measurement results (µm)

	Mean	SD	Max	Min
RMS	0.020	0.006	0.032	0.013
*P*	0.159>0.1			

*RMS* root mean square, *SD* standard deviation, *Min* minimum, *Max* maximum

The physical polyether cast (PPC) group was prepared with polyether impression material to simulate a tooth preparation (Fig. 2). Hydrophilic polyether material (Impregum Penta Soft; 3 M ESPE, St. Paul, MN, USA) was injected into the root canal. The material was subsequently applied to the prepared abutment structure to restore the buccolingual width and occlusogingival height, matching the adjacent teeth. This procedure aimed to replicate the original tooth dimensions prior to preparation using polyether material, thereby facilitating the acquisition of a digital model for crown preparation. Once completion of “tooth” preparation, a 3D cast was obtained using an intraoral scanner for the crown design. Then, the polyether post-and-core was extracted and scanned using a desktop scanner (D700; 3Shape, Copenhagen, Denmark) to construct its virtual cast. The digital cast was scaled down by 2.5% in Geomagic Wrap 2017 (Geomagic Wrap 2017; 3D Systems, Rock Hill, SC, USA) to get an 80-µm cement space around the post and the core [24]. The post-core, and crown were milled based on their corresponding digital cast.

The crowns were fabricated from composite resin blocks (Brilliant Crios; Coltène, Altstätten, Switzerland), while the post-and-cores were manufactured using fiber-reinforced composite resin blocks (cylindrical fibrous resin block; Eurasia Ruikang New Material Technology Co., Tianjin, China). All restorations were shaped immediately after milling without sintering and glazing. All procedures were performed by a dentist with technical training.

A high-resolution µCT device (NEMO NMC-100; PINGSENG Healthcare, Kunshan, China) evaluated the fit of the crowns. The average intracoronary cement thickness (AICT) and the linear gap between the crown and the abutment at selected sites were assessed. The µCT scanning was performed by rotating each specimen 360 degrees along its axis, with an average scan duration of approximately 30 min. The X-ray tube was operated at 90 kv and 60 µA with a voxel size of 0.8 μm. The acquired images were reconstructed using a software program (Avatar v1.6.5; PINGSENG Healthcare, Kunshan, China) for volumetric and linear calculations. A preliminary experiment verified the impermeability of both the resin bonding agent and the restoration under µCT analysis.

**Fig. 6:**
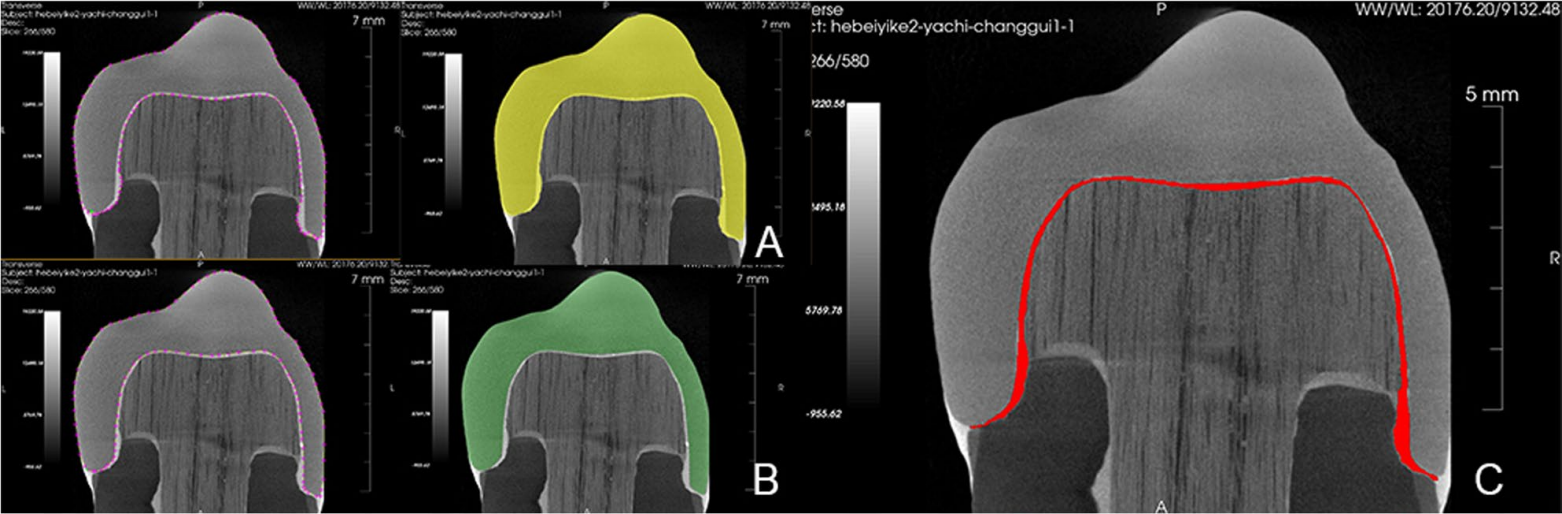
Diagram of the subtraction method for adhesive volume quantification. **A** Crown with adhesive (crown + adhesive) volume measurement; **B** Crown alone volume measurement; **C** Adhesive volume = **A** - **B**

The cement volume inside each specimen’s crown was obtained by rendering a 3D area with a growing grayscale range. Generally, the volume of an irregular object divided by its surface area is approximately equal to its average thickness. This study estimated the average intracoronary cement thickness (AICT) by dividing the volume of resin cement inside the crown by the surface area of the abutment (Fig. 6).

**Fig. 7:**
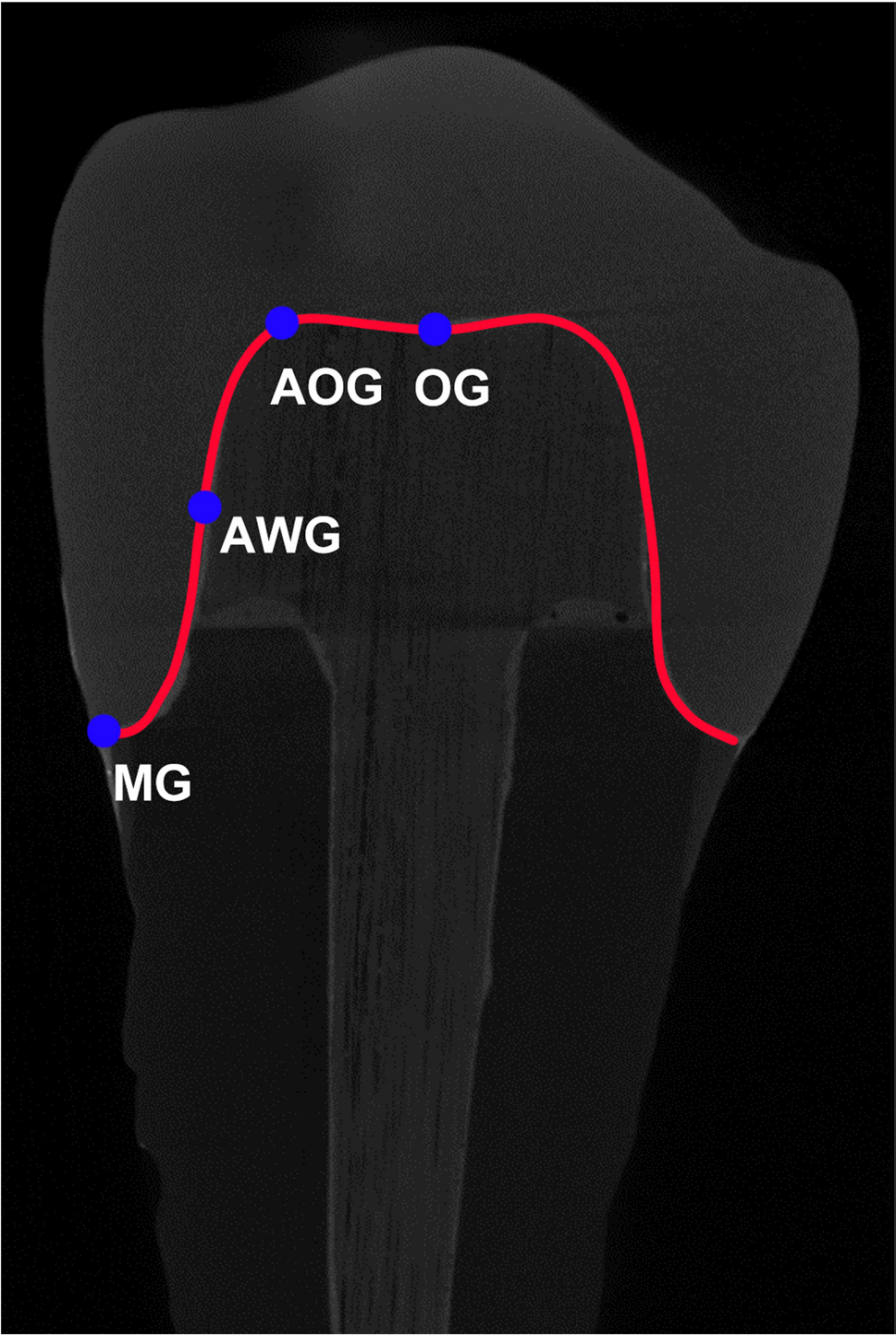
Schematic illustration of linear gap measurement sites between crown and abutment preparation. *AOG* Axial occlusal-plane intersection gap, *AWG* Axial wall gap, *MG* Marginal gap, *OG* Occlusal gap

The linear gap between the crown and abutment was measured in the sagittal and coronal planes. This measurement included four positional categories with eight points: 1 marginal gap (MG) and three internal gap points: the axial wall gap (AWG), the axial occlusal-plane intersection gap (AOG), and the occlusal gap (OG) (Fig. 7).

Statistical analysis was performed using IBM SPSS Statistics software (version 25.0; IBM, Armonk, NY, USA). Normality and homogeneity of variance were assessed using the Shapiro–Wilk and Levene’s tests, respectively. A significance level of α = 0.05 was used for all tests. For non‑normally distributed variables, data are presented as median (range) and were analyzed using the Kruskal–Wallis test, followed by post‑hoc pairwise comparisons with the Wilcoxon rank‑sum test with Bonferroni correction. For normally distributed variables, data are presented as mean ± standard deviation (SD). Variables meeting the assumption of homogeneity of variance (MG, AOG, OG) were analyzed using one‑way ANOVA with Tukey’s post‑hoc test. The variable AWG, while normally distributed, violated the homogeneity of variance assumption and was therefore analyzed using Welch’s ANOVA with the Games‑Howell post‑hoc test.

## Results

The median AICT values were 88 μm (range: 68–102 μm) for the TSS group, 92 μm (range: 53–171 μm) for the DSM group, and 99 μm (range: 74–140 μm) for the PPC group (Table 2). No statistically significant difference in AICT was observed among the three groups (*H* = 4.975, *P* = .083).

**Table 2:** Average intracoronary cement thickness (AICT) across groups, presented by median, interquartile range, minimum and maximum values. (AICT=volume/superficial area, µm)

	Median	IQR(Q1,Q3)	Min	Max
TSS	88	78–97	68	102
DSM	92	87–113	53	171
PPC	99	92–116	74	140
H	4.975			
*P*	.083			

*DSM* Digital cast splicing method, *PPC* Physical polyether cast method, *TSS* Traditional step-by-step workflow, *IQR* Interquartile range, *Min* Minimum, *Max* Maximum

**Fig. 8:**
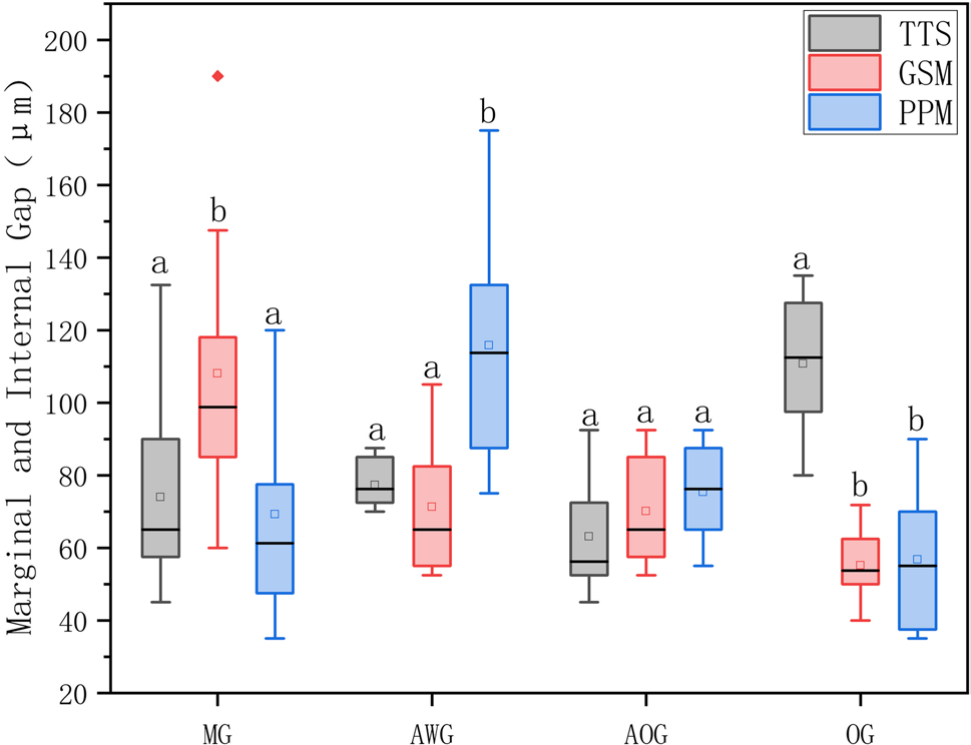
Marginal gap (MG) and internal gaps (AWG, AOG, OG) for each group. Same letters indicate no statistically significant difference at *P *≥ .05. No significant differences found for AOG among groups. Digital cast splicing method group exhibited significantly higher MG values than other groups. Physical polyether cast group had highest AWG values. TSS group showed significantly higher OG values than other 2 groups. *AOG* Axial occlusal-plane intersection gap, *AWG* Axial wall gap, *OG* Occlusal gap

As shown in Table 3; Fig. 8, the mean axial‑occlusal gap (AOG) did not differ significantly among the groups (*P* = .204). In contrast, statistically significant intergroup differences were observed for the following parameters:

- Marginal gap (MG) (*P* = .022): Post hoc analysis revealed that the DSM group (mean ± SD, 108 ± 38 μm) exhibited a significantly larger MG than both the TSS (mean ± SD, 74 ± 27 μm) and PPC (mean ± SD, 70 ± 29 μm) groups, which did not differ from each other.
- Axial wall gap (AWG) (*P* = .004): The PPC group (mean ± SD, 116 ± 34 μm) demonstrated a significantly larger AWG than both the TSS (mean ± SD, 77 ± 6 μm) and DSM (mean ± SD, 72 ± 18 μm) groups, with no significant difference between TSS and DSM.
- Occlusal gap (OG) (*P* < .001): The TSS group (mean ± SD, 111 ± 18 μm) had a significantly larger OG than both the PPC (mean ± SD, 57 ± 20 μm) and DSM (mean ± SD, 56 ± 10 μm) groups, which were comparable.

**Table 3:** Discrepancy values within 4 different regions of interest—MG, AWG, AOG, OG—across the 3 workflows ($\:\overline{x\:}$±s, µm)

	MG		AWG	AOG	OG
TSS	74 ± 27^a^	77 ± 6^a^		63 ± 16^a^	111 ± 18^a^
DSM	108 ± 38^b^	72 ± 18^a^		70 ± 15^a^	55 ± 10^b^
PPC	70 ± 29^a^	116 ± 34^b^		76 ± 13^a^	57 ± 20^b^
Statistic	(F) 4.418		(Welch’s F) 8.341	(F) 1.685	(F) 37.189
*P*	.022		.004	.204	< .001

In the “Statistic” row, “F” indicates One-Way ANOVA test applied for this group’s data, while “H” indicates Kruskal–Wallis test

*AOG* axial occlusal-plane intersection gap, *AWG* axial wall gap, *DSM* digital cast splicing method, *MG* marginal gap, *OG* occlusal gap, *PPC* physical polyether cast method, *TSS* traditional step-by-step workflow

Different lowercase letters within the same column indicate statistically significant differences (*P* < 0.05)

## Discussion

This study investigated the marginal and internal fit of crowns using three different workflows. These workflows included 2 simultaneous fabrication methods for custom-integrated fiber post-and-cores and crowns, along with a traditional workflow. A 2D and 3D comparative analysis was conducted to facilitate a more comprehensive evaluation. No significant differences were observed in the AOG and the AICT among the groups. However, significant differences emerged in the comparison of the MG, AWG, and OG between the 2 simultaneous digital fabrication workflows and the traditional workflow. The null hypothesis was rejected, as significant differences were observed in several marginal and internal fit parameters (MG, AWG, and OG) among the three workflows.

The adaptation of crown restorations directly influences the success of the post-core-crown restoration [38]. The clinically acceptable threshold for marginal adaptation is typically 120 μm [31], whereas the acceptable range for internal adaptation is still debated, with prior studies indicating 200–300 μm [39]. In this study, the average values of marginal and internal gap at linear measurement sites were less than 120 μm, which appears to meet clinical requirements. Compared to previous studies, the innovation of this research lies in the use of “cement volume/preparation surface area” to calculate the average intracoronary cement thickness (AICT). This approach avoids the impact of the preparation profile variation between different tooth morphologies, such as the mandibular second premolar (employed in this study) and the first molar (used in the previous study) [40]. The method presumes a uniform cement layer, thereby neglecting variations in its distribution across areas such as axial walls and occlusal surfaces. To overcome this limitation and verify its practical significance, we simultaneously measured the two-dimensional linear gaps at four locations. For example, the PPC group exhibited the largest median axial wall gap, indicating possible uneven distribution of cement in this area. It should be noted that even with multiple measurements, the “homogenization effect” of the average intracoronary cement thickness may still obscure site-specific adaptation risks to some extent. Further verification will require more precise measurement methods or clinical studies in the future.

The MG of a crown is particularly critical [41], and the horizontal discrepancy of the MG could be regulated during the fitting process; therefore, the vertical MG was measured. Three locations were selected for internal gap assessment: the axial wall, the intersection of the axial and occlusal surfaces, and the occlusal surface. Although statistically significant differences were observed among groups at certain measurement sites (MG, AWG, OG), all mean marginal and internal gap values were below 120 μm, indicating that these statistical differences do not affect clinical acceptability. Therefore, all three workflows meet clinical requirements [31].

The findings indicated that the OG in the TSS group was the largest. This may suggest that crowns in this group would require less adjustment before being seated. Given the current equipment, the 50 μm gap setting may be marginally excessive for the TSS group. This finding is consistent with previous studies indicating that CAD-CAM fabricated crowns tend to exhibit the largest OG [40]. The PPC group exhibited the largest axial wall gap (AWG) and the smallest marginal gap (MG). This discrepancy is attributed to an approximate 2.5% volumetric reduction of the polyether post-and-core analogue during its fabrication. Consequently, the final preparation morphology after analogue seating was effectively reduced, leading to a corresponding increase in the cement space (manifested as a larger AWG) between this reduced preparation and the crown. The crown seating also encountered less resistance, thereby allowing for a closer marginal fit. Also, the inability to polish the prepared polyether analogue likely introduced surface irregularities that were captured during scanning. This resulted in a morphological mismatch between the digitized analogue and the idealized digital crown design, ultimately contributing to the less uniform distribution of the cement layer observed along the axial walls in this group. Nevertheless, the measured bonding gap remained well below the permissible threshold of 300 μm that was reported by another study [39]. This trend suggests that the hybrid digital-analogue workflow may present challenges in achieving consistently uniform cement layer thickness, particularly in the axial region, despite delivering acceptable overall marginal adaptation.

The MG in the DSM group was the largest, at 108 μm, which may be related to inaccuracies in data superimposition or inherent systemic flaws associated with the operation and equipment. The DSM workflow, the primary innovation of this study, presents high technique sensitivity. Errors can be introduced during the software-based alignment and segmentation of the virtual models. Precisely identifying the identical point on both 3D surfaces with 100% reproducibility is inherently subjective and challenging. Thus, a critical challenge in the DSM workflow is the inability to achieve coplanar linear alignment between the virtual post-core and dentition models. Any resulting rotational deviation in the digital design translates directly into a seating discrepancy during the physical restoration assembly, which inevitably increases the marginal gap. Moreover, during the merging and processing of scan data, inaccuracies in matching point clouds accumulate and propagate to the restoration fabrication stage. It is important to note that the digital workflow incorporated a pragmatic clinical validation step. Although the initial digital registration was verified to be within an acceptable range (20 μm), final virtual designs were allowed minor manual refinements. This was essential to ensure the physical restorations achieved complete passive seating.

The choice of materials was strategically aligned with the study’s hypotheses and methods. Polyether was selected for the PPC workflow due to its superior hydrophilicity and flowability [42–43], which are critical for achieving the high-fidelity physical transfer of post-space morphology. This choice, however, inherently prevents the digital design of a controlled cement space. Thus, while enhancing crown fit fidelity through accurate morphology transfer, it directly results in the larger axial cement gaps observed. CAD-CAM resin composite crowns were predominantly chosen due to their superior edge durability against chipping during milling, absence of sintering requirements [44–45], ease of repair, and capability for same-day delivery.

Beyond statistical significance, the clinical relevance of the findings should be considered. Although the marginal gap values differed significantly among groups, all three workflows produced crowns with marginal gaps below the clinically acceptable threshold of 120 μm. This suggests that all workflows—including the conventional method—are capable of producing clinically acceptable restorations. The digital workflows, on the other hand, have a clinically significant benefit because they make treatment more efficient by being more accurate and allowing fewer patient visits without lowering quality.

The main strengths of this study include (1) the use of a comprehensive digital workflow for simultaneous fabrication of integrated post-and-core and crowns, which reflects a clinically relevant scenario; and (2) the combined use of 2D and 3D measurement methods, providing a comprehensive assessment of restoration fit. Several limitations should be acknowledged. First, the sample size of 10 specimens per group, while comparable to previous in vitro studies, is relatively modest; larger sample sizes would enhance statistical robustness. Second, the anatomical variations in root and canal morphology among the premolars were not strictly controlled. Although post adaptation was not assessed, such variations may still influence crown seating and cement-space distribution, representing a potential confounding factor. Third, the experiment was conducted under ambient conditions without simulating intraoral temperature and humidity, which may affect the accuracy of impression materials and thus overestimate adaptation in conventional workflows. Additionally, the crowns were seated using manual finger pressure without objective quantification of the applied force or duration, which may have introduced variability in cement thickness and marginal adaptation. This in vitro study does not reflect long-term clinical performance, as factors such as cement degradation can alter restoration adaptation over time [46]. Future clinical studies are warranted to validate these findings.

## Conclusion

Within the limitations of this in vitro study, the digital workflows represent a reliable alternative to conventional multi-visit protocols for fabricating integrated post-and-core and crowns. The consolidation of the procedure into a single visit addresses a key clinical concern—minimizing treatment burden—without compromising restoration fit. 

## Data Availability

All essential data is presented in the manuscript. The datasets and images are available from the corresponding author on reasonable request.

## Abbreviations

CAD-CAM: Computer-aided design and computer-aided manufacturing
PEEK: Polyetheretherketone
µCT: Microcomputed tomography
TSS: Traditional step-by-step workflow
DSM: Digital cast splicing method
PPC: Physical polyether cast
MG: Marginal gap
AWG: Axial wall gap
AOG: Axial occlusal intersection gap
OG: Occlusal gap
AICT: Average intracoronary cement thickness
